# A Scene-Aware Degradation Universal Re-Identification Framework for Adverse Weather

**DOI:** 10.3390/s26102951

**Published:** 2026-05-08

**Authors:** Siwei Wei, Yuxin Wang, Mingxuan Yang, Chunzhi Wang

**Affiliations:** 1 School of Computer and Artificial Intelligence, Wuhan University of Technology, Wuhan 430070, China; waosfengw@whut.edu.cn (S.W.); wyx@whut.edu.cn (Y.W.); 135747@whut.edu.cn (M.Y.); 2 School of Computer Science, Hubei University of Technology, Wuhan 430068, China

**Keywords:** re-identification, scene semantics awareness, feature decoupling, CLIP, adverse weather

## Abstract

**Highlights:**

**What are the main findings?**

- The proposed ScA-UniReID framework, built upon CLIP, effectively addresses the challenge of ReID under coupled adverse weather (e.g., rain and fog) by dynamically disentangling identity semantics from degradation artifacts using dual textual prompts and an adaptive control module.

**What are the implications of the main findings?**

- This work provides a novel cross-modal paradigm that moves beyond conventional image-enhancement or robust-feature approaches, offering a new solution for ReID in complex, real-world environments where multiple degradations co-occur.

**Abstract:**

Vision-based Re-identification (ReID) is crucial for intelligent surveillance yet remains vulnerable to adverse-weather degradations such as rain and fog, which simultaneously corrupt visual clarity and identity-specific cues. Existing image-enhancement and robust-feature paradigms struggle when multiple degradations co-occur, while recent CLIP-based ReID models have scarcely examined cross-modal alignment under weather distortions. To bridge this gap, we propose ScA-UniReID, a Scene-Aware Degradation Universal ReID framework built upon CLIP’s dual-encoder architecture. ScA-UniReID introduces dual textual prompts—target-oriented for identity features and degradation-oriented for weather noise—and an adaptive control module that dynamically re-weights them to disentangle identity semantics from degradation artifacts. Extensive experiments on pedestrian and maritime ReID benchmarks under diverse adverse-weather protocols show that ScA-UniReID outperforms state-of-the-art methods and generalizes robustly to unseen conditions, validating its efficacy and universality.

## 1. Introduction

Target re-identification (ReID) aims to retrieve and recognize specific individuals across large-scale image databases captured by different cameras and over extended periods [1]. As a cornerstone of intelligent perception, it underpins a wide range of applications—from smart transportation, public security [2], and maritime surveillance [3] to smart retail and urban management. Yet, the performance of any vision-based recognition system is fundamentally limited by image clarity and content integrity [4]. In real-world complex environments, images are frequently degraded by noise, motion blur, and adverse weather conditions such as rain, fog, or low-light scenarios, which significantly hinder the practical effectiveness of ReID systems.

To counteract the adverse effects of degraded images, existing research has generally pursued two complementary technical avenues [5,6]. The first operates at the input level: image-enhancement pipelines that denoise, dehaze, or super-resolve imagery before it reaches the recognition network [7,8]. The second avenue focuses on the model level: designing more robust feature extractors [9,10] that learn identity representations resilient to various image corruptions. Despite notable successes, empirical studies reveal that both strategies suffer limited generalization when multiple degradation factors co-occur—such as heavy rain at night—because they struggle to simultaneously restore visual clarity and preserve identity-specific cues.

In recent years, the rise of Vision-Language Pre-training (VLP) has provided new solutions for re-identification research. Cross-modal models such as CLIP (Contrastive Language-Image Pre-training) [11] employ large-scale image-text contrastive learning to jointly optimize visual and text encoders, mapping multimodal data into a unified semantic embedding space. This enables the alignment of semantically relevant samples and the separation of irrelevant ones, thereby modeling high-level semantic correspondences across modalities, and achieves strong semantic alignment capability and excellent zero-shot transfer performance [12]. Within the ReID community, researchers have begun to exploit learnable textual prompts to steer visual encoders toward identity-relevant regions, thereby enhancing fine-grained discriminative power [13,14]. However, most CLIP-based ReID studies have concentrated on standard, well-controlled scenarios. They have yet to systematically investigate how rain, fog, or other degradation factors distort cross-modal alignment, leaving models vulnerable in complex, real-world environments [15,16].

To address the limitations mentioned above, this paper proposes a Scene-Aware Degradation Universal ReID framework (SCA-UniReID). Built upon the dual-encoder architecture of CLIP [17], the proposed method introduces a scene-aware degradation modeling mechanism to explicitly characterize environmental factors such as rain, fog, and low-light conditions. Specifically, we develop a Scene-Aware Degradation CLIP (SCA-CLIP), which incorporates a scene perceiver to learn degradation-aware representations and guide the visual encoder to focus on identity-relevant features under adverse conditions.

Furthermore, we design a dual-textual semantic guidance mechanism consisting of a target-oriented prompt and a scene-aware prompt. The former enhances identity-discriminative information, while the latter adaptively captures scene-specific degradation characteristics. An adaptive control module dynamically balances the contributions of the two prompts, enabling effective disentanglement of identity semantics from degradation noise (i.e., decomposing the latent representation into interpretable and minimally correlated subspaces). This design allows the model to achieve robust cross-modal alignment while accounting for environmental interference, thereby significantly improving ReID performance under complex, real-world conditions.

- We systematically analyze the challenges posed by rain-and-fog coupled degradations to existing ReID systems and reveal the limitations of both image-enhancement and robust-feature paradigms under extreme weather.
- We propose SCA-UniReID, a scene-aware universal ReID framework that integrates dual textual prompts—target-oriented and degradation-oriented—into a CLIP-style dual-encoder architecture, enabling fine-grained disentanglement of identity semantics from weather noise while preserving discriminability.
- We conduct comprehensive experiments on ship and pedestrian benchmarks under multiple adverse-weather protocols; results demonstrate that SCA-UniReID surpasses state-of-the-art methods and maintains strong generalization across unseen conditions.

## 2. Related Works

### 2.1. Image Preprocessing Methods in Re-Identification

Complex environments can cause image degradation, making it difficult for ReID models to extract stable identity features. Image enhancement methods aim to improve image quality through preprocessing, enabling better visibility in complex environments, thus improving feature extraction performance. Jiao et al. [18] combined super-resolution convolutional networks with ReID networks to enhance the re-identification performance of low-resolution images. To further improve the scale adaptability of super-resolution methods, Wang et al. [19] adopted a cascaded SRGAN structure, progressively reconstructing missing details to improve the super-resolution techniques’ ability to adapt to different scales. Zhang et al. [20] proposed a frequency-domain modeling framework based on wavelet decomposition, which enhances detail recovery capability through multi-scale feature decomposition and makes the feature distribution conform to the atmospheric scattering model. In addition, Liu et al. [21] proposed a prior model based on inverse haze density correction, which models the transmittance via pixel-level gamma correction, improving the generalization performance of the method in various degraded scenarios. Mao et al. [22] proposed the FFSR module and designed a dual-branch module to extract resolution-invariant features, further optimizing the detailed representation of target re-identification. Huang et al. [23] proposed a pedestrian re-identification framework that addresses illumination changes. By utilizing the Retinex theory for illumination decomposition, they designed a bottom-up attention network aimed at eliminating interference in low-light environments. In real-world surveillance scenarios, adverse weather conditions such as rain and haze can significantly degrade image quality, thereby affecting the extraction of discriminative features for person re-identification (ReID). To address this issue, existing studies have explored joint deraining and dehazing methods toenhance the visibility of degraded images. For example, Ragini et al. [24] proposed a Single-Stage V-Shaped Network (S2VSNet) for end-to-end image deraining and dehazing. Similarly, Xie et al. [25] combined the dark channel prior (DCP) with deep learning models, further improving image restoration performance through atmospheric light modeling. Although these methods primarily focus on low-level visual enhancement, they effectively reduce environmental interference and provide more reliable inputs for subsequent high-level tasks. Despite the progress of these methods under degraded conditions, they still have limitations. Since image enhancement is merely a preprocessing step for the ReID task, it is somewhat disconnected from the target recognition task. The enhanced image may have domain shifts compared to the real high-quality image, leading to feature extraction and matching biases, which ultimately affect recognition performance.

### 2.2. Discriminative Feature Learning Methods in Re-Identification

Different from strategies centered on image enhancement, such technical routes target the ReID task for direct optimization, allowing feature extractors to steadily capture identity-related representations within complicated scenes, and bypassing the risks of information missing and domain migration that may be triggered by image enhancement operations [26]. At present, relevant studies are mainly split into two branches: the first is degradation-robust learning via feature decoupling, and the second is end-to-end modeling driven by multi-task synergistic optimization [27].

The former branch (degradation-robust learning via feature decoupling) is designed to strengthen the environmental adaptability of ReID models in complex scenarios. Huang et al. [28] put forward a degradation-agnostic learning architecture that extracts identity features while suppressing degradation interference in an unsupervised manner. Zeng et al. developed a dual-branch network structure to offset the disturbance of illumination fluctuations on identity characterization for all-weather application scenes. Moreover, to quantitatively evaluate the anti-degradation performance of conventional ReID models, Chen et al. [29] built a specialized benchmark dataset for degradation robustness testing in object re-identification, and further established a universal ReID baseline with strong generalization capability on this basis. Kanwal et al. [30] pioneered a feature integration framework that combines dark channel prior knowledge with transfer learning, merging global semantic features and local prior clues to elevate the robustness of object re-identification systems.

Joint optimization-driven end-to-end schemes strive to boost image clarity and recognition precision synchronously via shared feature representation and multi-task collaborative learning [31]. For pedestrian re-identification tasks, Zheng et al. [32] tackled the cross-resolution matching challenge by proposing a bilateral resolution-aware identity modeling approach. Lu et al. presented an Illumination Distillation Framework (IDF) that integrates illumination enhancement and knowledge distillation mechanisms, enhancing the model’s adaptability to low-light environments and lifting nighttime ReID accuracy effectively.

In the vehicle re-identification domain, Chen et al. [33] designed a Semi-supervised Joint Defogging Learning (SJDL) framework for foggy-scene ReID tasks. This framework realizes end-to-end cooperative training of defogging and re-identification, and shares degradation-free features across tasks to eliminate domain shift problems existing in traditional two-step pipelines. Besides, the research team further proposed the RVSL training paradigm [34], which fulfills vehicle ReID without relying on manual annotations and clean reference images. For ship re-identification, most existing methods are only applicable to calm sea conditions, whereas the marine environment features strong randomness and is frequently affected by harsh factors like high humidity, heavy fog and low visibility. Yasod et al. [35] developed a thermal imaging-based ship monitoring solution, which is distinctive for conducting fine-grained local feature matching via ship contour information instead of employing standard re-identification pipelines.

### 2.3. Vision-Language Learning

In recent years, the Vision-Language Pretraining (VLP) paradigm, particularly the introduction of CLIP (Contrastive Language-Image Pretraining), has significantly advanced multimodal representation learning between images and language. CLIP establishes a deep connection between visual content and natural language through contrastive learning on large-scale image-text pairs, demonstrating strong transferability across various domains. In this “pretraining and fine-tuning” paradigm, the quality of the pretrained model plays a crucial role in the optimization difficulty and performance of downstream tasks. Fine-tuning methods based on prompts or adapters have been widely applied in the vision domain. CLIP-Adapter [13] adds a lightweight adapter module on top of CLIP’s image and text encoders to improve task-specific fine-tuning effectiveness. Li et al. [14] first proposed the CLIP-ReID model, using learnable prompts to guide the visual encoder to extract more semantically rich image features. Yu et al. [12] further introduced the CLIP-driven Semantic Discovery Network, which partially addresses the semantic consistency issue between modalities. Based on the above research, we aim to further utilize CLIP’s vision-text representation capability to extract degradation characteristics of complex scenes, thereby advancing the development of the object re-identification field.

## 3. Preliminaries

In this section, we first review CLIP, followed by the introduction of the proposed Scene-Aware CLIP and Scene-Aware Prompts.

### 3.1. CLIP Framework

CLIP is a large-scale pretrained vision-language model composed of two encoders: the image encoder *I(·)* and the text encoder *T(·)*. The image encoder *I(·)* is mainly based on two backbone networks, ResNet-50 and ViT-B/16, while the text encoder *T(·)* is implemented using Transformer blocks. In classification tasks, CLIP adopts a simple yet effective method to construct text prompts, usually using templates like “*A photo of a [CLS]*,” where [CLS] is replaced with specific class names. On this basis, CoOp [36] introduces learnable text contexts, represented as “*[v]*_1_*[v]*_2_*...[v]_m_[CLS]*.” This method dynamically generates meta-tokens based on different input images and, by combining learnable context vectors, enhances the model’s transferability and performance. By leveraging the capabilities of CLIP and CoOp, deeper semantic understanding can be achieved, allowing for more robust and accurate modeling in re-identification tasks under complex scenarios. Given an image, CLIP calculates the similarity between the image embedding and the text prompt embedding. The entire process involves projecting both the image and text into a shared high-dimensional space for quantitative evaluation of their relationship. The specific objective function is as follows:

(1) $$s\left(V_{i},T_{i}\right)=V_{i}\cdot T_{i}=g_{v}({img}_{i})\cdot g_{T}({text}_{i})$$

(2) $$L_{i2t}\left(i\right)=-log\frac{exp\left(s\left(V_{i},T_{i}\right)\right)}{\sum_{k=1}^{N}exp\left(s\left(V_{i},T_{k}\right)\right)},\;L_{t2i}\left(i\right)=-log\frac{exp\left(s\left(V_{i},T_{i}\right)\right)}{\sum_{k=1}^{N}exp\left(s\left(V_{k},T_{i}\right)\right)}$$

In this context, $g_{V}\left(\cdot \right)$ and $g_{T}\left(\cdot \right)$ are linear layers that project the embeddings into the cross-modal embedding space. $L_{i2t}\left(i\right)$ represents the image-to-text contrastive loss, while $L_{t2i}(i)$ represents the text-to-image contrastive loss.

### 3.2. Scene-Aware-Degradation Module

The Scene-Aware Degradation (SCA) module is a copy of the CLIP image encoder that introduces a small number of zero-initialized connection modules to dynamically control the image encoder, optimizing its feature extraction ability in degraded environments. The core function of SCA is to adjust the output of the image encoder, guiding the model to focus on the target features and suppressing the influence of noise from weather-related degradations (such as rain or fog) on recognition performance.

This study designs corresponding control modules for two different backbone networks: ResNet-50 and ViT-16. In the ResNet-50 structure, the Scene-Aware module uses the output of each layer as a hidden control signal. After passing through the zero-initialized connection modules, the control signal is added to the target encoder. The initialization module consists of convolutional layers, normalization layers, and ReLU functions, with all parameters initially set to zero to ensure that the early stages of training do not interfere with the extraction of target features. As training progresses, the control signal gradually adjusts the behavior of the encoder, focusing more on the target area and ignoring the interference from degradation factors.

In the ViT-16 structure, the control signal comes from the output of the Transformer blocks. These outputs are then combined with relevant layers of the target encoder. By adding the control signal, the predictions are adjusted. The Transformer blocks are connected by a simple fully connected neural network, with these connections also using a zero-initialization strategy to allow for gradual weight adjustments during training, adapting to different environmental conditions.

### 3.3. Scene-Aware Prompts

**Figure 1:**
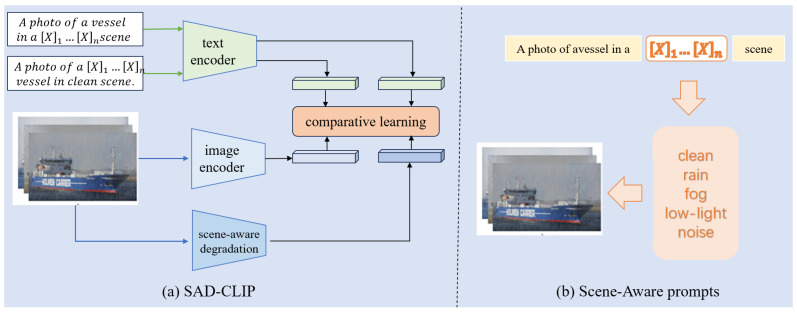
ScA-CLIP: (**a**) Scene-aware degradation learning in SAD-CLIP; (**b**) Scene-aware prompt generation.

In the CLIP model, traditional fixed prompts struggle to accurately characterize complex degradation patterns such as rain, fog, and low-light conditions, and fail to adapt to the regional variability of degradation features. To address this, we propose the ScA-CLIP scene-aware prompt learning mechanism: as shown in Figure 1b, we take degradation types such as clean, rain, and fog as learnable prompts to construct dynamic scene prompt templates; as illustrated in Figure 1a, we use comparative learning to align degraded scene prompts with degraded image features, and clean scene prompts with clean image features within the CLIP embedding space. This not only strengthens the model’s ability to perceive degraded regions but also enables the model to adapt to various adverse weather scenarios without additional annotations, effectively resolving the issue of insufficient adaptability caused by dataset limitations, while reducing the interference of high-level semantic information on the scene-aware module.

The design of the scene-aware prompt is as follows:

(3) $${prompt}_{sc}=A\;photo\;of\;a\;vessel\;in\;a\;{\left[X\right]}_{1}\ldots {\left[X\right]}_{n}scene$$

In this context, ${\left[X\right]}_{i}(i=1,2\ldots ,n)$ represents randomly initialized learnable labels that learn different degradation factors of the image. This format of the prompt is capable of learning various scene features and adapting to multiple coupled degradation factors, such as rain, fog, low light, etc. As a result, the model takes into full consideration the impact of environmental factors on the target features when performing cross-modal feature alignment.

## 4. Implementation Method

**Figure 2:**
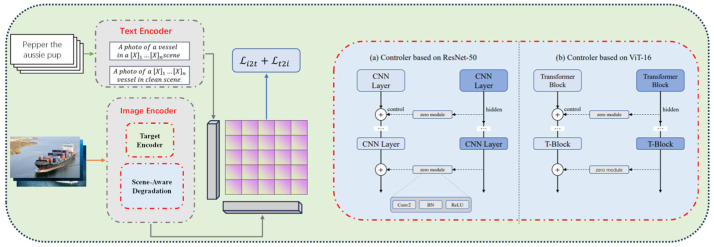
Overview of ScA-UniReID. Our framework comprises: (**a**) Construction of Adaptive Scene-Aware Perceptron and Controller on ResNet-50 and ViT-16; (**b**) text encoder based on Scene-Aware Prompts.

In this section, we introduce the core idea of our scene semantic-aware feature decoupling network, which aims to separate target vessel semantics from complex, degraded scene interference to enhance visual perception robustness under adverse weather. The network consists of two core stages:

First, semantic prompt construction and feature guidance, as illustrated in Figure 2, where we build learnable scene-aware prompts (e.g., clean, rain, fog) to achieve contrastive alignment between text and degraded/clean image features in the CLIP embedding space, guiding the model to focus precisely on degraded regions;

Second, target-scene encoding and feature decoupling, as illustrated in the figure, the image encoder is split into a target encoder and a scene-aware degradation module, paired with two controller designs based on ResNet-50 and ViT-16 to dynamically regulate feature flow, effectively decoupling target features from scene degradation features while mitigating interference from high-level semantic noise.

### 4.1. Overview of the Scene Semantic-Aware Feature Decoupling Network Framework

Building upon the previously proposed scene-aware CLIP, this section further develops the scene semantic-aware feature decoupling method, ScA-UniReID, aimed at enhancing the model’s adaptability to different target categories and rain/fog environments. The framework integrates cross-modal semantic guidance with a target-scene decoupling strategy to optimize feature modeling capabilities. This enables the model to precisely extract identity features even under varying degradation conditions, effectively suppressing degradation interference and improving the model’s generalization in rainy or foggy environments.

Specifically, ScA-UniReID uses a two-stage training strategy. In the first stage, the text encoder is trained to optimize the text-image alignment ability through adjustable prompts, allowing the model to adapt to different degradation environments and improving its semantic understanding of identity features. This provides more accurate guidance for subsequent target feature learning. In the second stage, the target encoder and the scene-aware module are jointly trained. The scene-aware module serves as an auxiliary branch and uses a control mechanism to guide the target encoder’s focus on identity information, thereby enhancing the model’s feature extraction stability under various degradation conditions. The training process is as shown in Algorithm 1.
**Algorithm 1** Training Process of Scene Semantic-Aware Feature Decoupling Network Framework**Stage 1: Load Pre-trained Models** $I_{id}\left(\cdot \right)$, $I_{sc}\left(\cdot \right)$, $T\left(\cdot \right)$, **Initialize Learnable Parameters** **Output:** Learnable Text Prompts 1.  for epoch=1…M do 2.    The target encoder $I_{id}\left(\cdot \right)$ and the scene-aware module $I_{sc}\left(\cdot \right)$ extract image features respectively 3.    Define dual text learnable prompts as shown in Equation (3) 4.    The text encoder $T\left(\cdot \right)$ encodes the dual text as shown in Equation (4) 5.    Update the prompts via backpropagation as shown in Equation (7) 6.  End for**Stage 2: Load Pre-trained Models** $I_{id}\left(\cdot \right),I_{sc}\left(\cdot \right),T\left(\cdot \right)$, **Dual Text Prompts** ${prompt}_{id}\;and\;{prompt}_{sc}$ **Output:** Trained Target Encoder $I_{id}\left(\cdot \right)$ and Scene−Aware Module $I_{sc}\left(\cdot \right)$ 7.  for epoch=1…N do 8.    The text encoder T(⋅) extracts text features from the dual text prompts 9.    The target encoder $I_{id}\left(\cdot \right)$ and scene-aware module $I_{sc}\left(\cdot \right)$ extract image features respectively 10.     Update the parameters of $I_{id}\left(\cdot \right)\;and\;I_{sc}\left(\cdot \right)$ via backpropagation (9) 11.     Fix the parameters of $I_{sc}\left(\cdot \right)$ and update $\;I_{id}\left(\cdot \right)\;$ via backpropagation (10) 12.   End for

### 4.2. Semantic Prompt Construction and Feature Guidance

**Figure 3:**
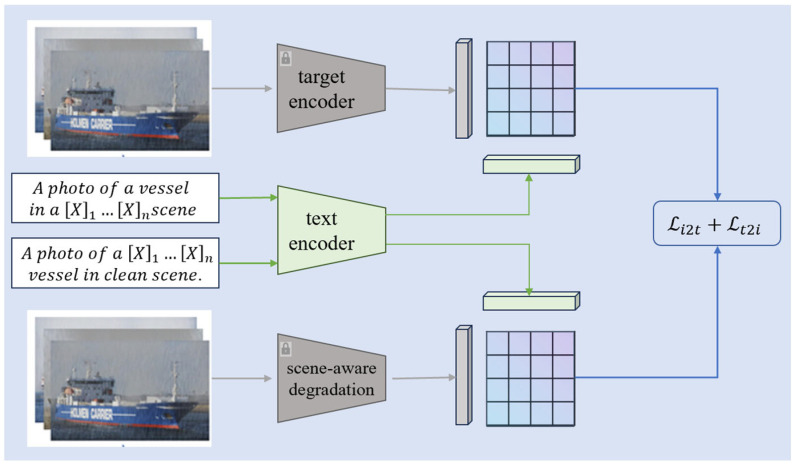
Framework of the First Stage Training of the Text Encoder for ScA-UniReID: Dual Prompting and Scene-Aware Feature Alignment.

In complex scenarios, target identity features are highly coupled with mixed rain and fog noise, making traditional fixed text prompts unable to model both target semantics and degradation patterns precisely. To address this, the first stage of ScA-UniReID (whose overall framework is shown in Figure 3) adopts a dual-text prompting mechanism and a dedicated scene-aware degradation pipeline:

As illustrated in Figure 3, we construct target-scene dual-text prompts by inserting learnable degradation tokens (e.g., rain, fog) into a CLIP-compatible prompt template. This design explicitly decouples semantic modeling: the target prompt guides the frozen target encoder to extract clean vessel identity features, while the scene-aware prompt drives the Scene-Aware Degradation Module—a parallel branch encoder that extracts low-level degradation features (e.g., raindrop textures, fog scattering) and aligns them with degradation semantics in the CLIP embedding space. To further regulate feature flow and avoid semantic interference, we introduce a lightweight controller module that dynamically gates the propagation of target and scene features in the backbone, ensuring effective decoupling of identity and degradation signals.

Via the contrastive loss $L_{i2t}+L_{t2i}$, we align the target encoder features with the clean text prompt and the scene module features with the degradation text prompt in the CLIP embedding space. Experimental results validate the effectiveness of these components: on the VesselReID_Adverse and Market_Adverse datasets, ScA-UniReID outperforms the baseline CLIP-ReID and other state-of-the-art methods, achieving 63.2% mAP and 75.9% Rank-1 accuracy on VesselReID_Adverse, with consistent improvements across both ResNet-50 and ViT-16 backbones. This confirms that the collaborative design of dual prompting, the scene-aware degradation module, and the controller enables the model to dynamically adapt to diverse degradation conditions while effectively separating target identity from noise, thus significantly enhancing recognition performance.

In the first stage, to effectively utilize the CLIP text encoder, this paper designs target-scene dual-text prompts, which are used to model target identity information and rain-fog degradation information, in order to enhance text-image alignment ability. The target text prompt and scene-aware prompt are defined as follows:

(4) $${prompt}_{id}=A\;photo\;of\;{\left[X\right]}_{1}\ldots {\left[X\right]}_{n}\;vessel\;in\;clean\;scene.$$

(5) $${prompt}_{sc}=A\;photo\;of\;{a\;vessel\;in\;a\;\left[X\right]}_{1}\ldots {\left[X\right]}_{n}\;scene.$$

Using the pre-trained identity encoder $I_{id}\left(\cdot \right),\;scene\;encoder\;I_{sc}\left(\cdot \right)$ and text encoder $T\left(\cdot \right)$, we extract the target identity feature $V_{id}$ and $V_{sc}$ and bilingual semantic text features. During the training phase, by freezing the parameters of $I_{id}\left(\cdot \right)$ and $I_{sc}\left(\cdot \right)$, we focus on optimizing the text tokens to learn contextual representations, thereby obtaining a unique textual representation for each identity (ID) and its corresponding scene. The formulation is as follows:

(6) $$T_{id}=T({prompt}_{id})$$

(7) $$T_{sc}=T\left({prompt}_{sc}\right)$$

Finally, based on the principle of Equation (1), the image-text contrastive objective function is defined as follows:

(8) $$L_{i2t}\left(i\right)=-log\frac{exp\left(s\left(V_{id}^{i},T_{id}^{i}\right)\right)}{\sum_{k=1}^{N}exp\left(s\left(V_{id}^{i},T_{id}^{i}\right)\right)}-log\frac{exp\left(s\left(V_{sc}^{i},T_{sc}^{i}\right)\right)}{\sum_{k=1}^{N}exp\left(s\left(V_{sc}^{i},T_{sc}^{i}\right)\right)}$$

In this principle, $L_{i2t}$ denotes the image-to-text contrastive loss, $L_{t2i}$ denotes the text-to-image contrastive loss, and $S\left(\cdot \cdot \right)$ represents the similarity function. N is the batch size. Since multiple images in a single batch may belong to the same identity, this implies that there may be multiple positive samples. Therefore, the computation of the text-to-image contrastive objective function is as follows:

(9) $$L_{t2i}\left(y_{i}\right)=-\frac{1}{\left|P\left(y_{i}\right)\right|}\sum_{p\in P\left(y_{i}\right)}log\frac{exp\left(s\left(V_{id}^{p},T_{id}^{y_{i}}\right)\right)}{\sum_{k=1}^{N}exp\left(s\left(V_{id}^{k},T_{id}^{y_{i}}\right)\right)}\frac{1}{\left|P\left(y_{i}\right)\right|}\sum_{p\in P\left(y_{i}\right)}log\frac{exp\left(s\left(V_{sc}^{p},T_{sc}^{y_{i}}\right)\right)}{\sum_{k=1}^{N}exp\left(s\left(V_{sc}^{k},T_{sc}^{y_{i}}\right)\right)}$$

Therefore, the final objective function for the first-stage training is as follows:

(10) $$L_{\mathrm{stage}1}=L_{i2t}+L_{t2i}$$

### 4.3. Target-Scene Encoding and Feature Disentanglement

**Figure 4:**
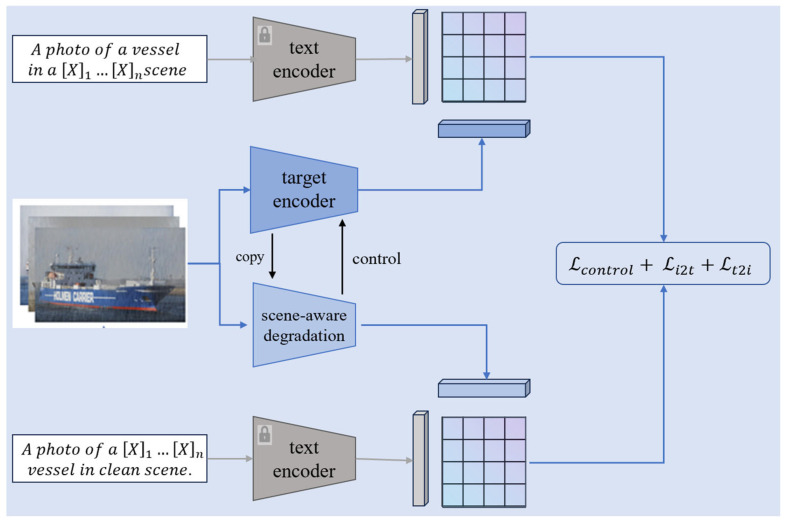
Framework Diagram of Target-Scene Encoding and Feature Disentanglement in the Second Stage.

In the second stage, we use an identity encoder and a scene encoder for contrastive learning to effectively disentangle target identity features from rain/fog noise (whose overall framework is shown in Figure 4). The scene encoder is initialized by copying the identity encoder, and then zero-initialized layers are added to the intermediate layers to enable conditional interactive learning with the identity encoder. The scene encoder continuously learns rain/fog degradation features from images, generating an implicit representation vector of the noise, which is then fed into the identity encoder as a conditional signal. Contrastive learning is performed between the two encoders to guide the identity encoder to reduce its focus on noisy regions and enhance its attention to the target identity feature regions.

In our study, contrastive learning objective function is employed to ensure effective alignment of image features and text descriptions in the embedding space. The objective function is defined as follows:

(11) $$L_{con}\left(V,T\right)=-\frac{1}{N}\sum_{i=1}^{N}q_{i}log\frac{exp\left(s\left(V^{i},T^{i}\right)\right)}{\sum_{j=1}^{N}exp\left(s\left(V^{j},T^{j}\right)\right)}$$

*N* represents the number of paired embeddings in a training batch, and $q_{i}$ denotes label smoothing. The optimization goal of this function is to maximize the cosine similarity between correctly paired text-image embeddings, while increasing the distance from incorrectly paired samples, enabling degraded images to find the most matching textual descriptions in the embedding space.

To jointly optimize the target identity features and noise features, this section further defines a combined objective function. By using the scene encoder to conditionally control the identity encoder, the latter treats noisy regions as negative samples and target feature regions as positive samples, thereby enhancing its focus on the desired target regions. The objective function is formulated as follows:

(12) $$L_{\mathrm{control}}=L_{\mathrm{con}}\left(V_{id},T_{id}\right)+L_{\mathrm{con}}\left(V_{sc},T_{sc}\right)$$

$V_{id}$ and $V_{sc}$ denote the target identity features and noise features extracted from the original image by the identity encoder $I_{id}\left(\cdot \right)$ and the scene encoder $I_{sc}\left(\cdot \right)$ respectively. Meanwhile $T_{id}$ and $T_{sc}$ are the textual representation vectors obtained from the target text and scene-aware prompt words using the text encoder $T\left(\cdot \right)$ trained in the first stage.

Furthermore, to enhance the adaptation of the target encoder to the target re-identification task, the cross-entropy loss $L_{id}$ and the triplet loss $L_{tri}$ are further employed to optimize the identity encoder:

(13) $$L_{id}=\sum_{i=1}^{N}-q_{i}log\left(p_{i}\right)$$

(14) $$L_{tri}=max\left(d_{p}-d_{q}+\alpha ,0\right)$$

$q_{i}$ denotes the true label of the *i*-th sample, and $p_{i}$ is the predicted probability of the true label. $d_{p}$ and $d_{q}$ represent the feature distances of the positive and negative sample pairs, respectively, while α is the margin parameter of the triplet loss $L_{tri}$. The final objective function for the second stage is as follows:

(15) $$L_{\mathrm{stage}2}=\lambda_{1}L_{\mathrm{control}}+\lambda_{2}\left(L_{id}+L_{tri}\right)$$

## 5. Experiment

To evaluate the performance of the proposed ScA-UniReID method in target re-identification tasks, we conduct experiments on both ship and pedestrian datasets and compare our approach with state-of-the-art methods in the field of person and object re-identification. Furthermore, to deeply analyze the contribution of each module to the overall model performance, ablation studies are carried out in this section to investigate the impact of different modules and parameters on the model’s effectiveness. Finally, visualization analyses are provided to demonstrate the recognition performance of different methods under degraded conditions, making the experimental conclusions more intuitive and interpretable.

### 5.1. Experimental Settings

In the training phase, we adopt modified versions of ResNet-50 and ViT-16 pretrained on CLIP as the backbone networks for feature extraction. The Adam optimizer is used during training, along with data augmentation techniques such as random horizontal flipping, cropping, and erasing. A global attention pooling layer reduces the feature dimension from 2048 to 1024; correspondingly, the text feature dimension is scaled from 512 to 1024 for alignment. For the ViT-16 backbone, the batch size is set to 32, image size to 384 × 256, and the feature dimension is reduced from 768 to 512, while the text feature dimension remains at 512. Additionally, on the VesselReID_Adverse dataset, the batch size is set to 64 and the image size to 384 × 192. Since pedestrian images are generally smaller, following the AGW [37] protocol, the batch size is set to 64 and the image size to 256 × 128 on the Market_Adverse dataset.

In the first training stage, two textual prompt tokens are trained for 60 epochs on each dataset, with an initial learning rate of 3.5 × 10^−4^, which is adjusted using a cosine annealing scheduler. In the second training stage, the identity encoder and scene encoder use the ResNet-50 backbone and are trained for 120 epochs on the dataset, with an initial learning rate of 3.5 × 10^−4^. The learning rate is reduced to one-tenth of its current value at the 40th and 70th epochs. When using the ViT-16 backbone, the model is trained for 60 epochs with an initial learning rate of 5 × 10^−6^, and the learning rate is similarly reduced at the 30th and 50th epochs.

Moreover, to better adapt the network to the target re-identification task when using the ResNet-50 backbone, the two encoders are trained synchronously for the first 60 epochs. Then, in the subsequent 60 epochs, the scene encoder is frozen, and the identity encoder is optimized using Equations (10) and (11).

### 5.2. Comprehensive Experimental Comparison and Analysis

To comprehensively evaluate the performance of the proposed ScA-UniReID method in the task of person re-identification under rainy and foggy conditions, we conducted extensive experiments on the VesselReID_Adverse and Market_Adverse datasets, comparing it with the baseline model CLIP-ReID [14] and several state-of-the-art re-identification methods, including AGW, TransReID [38], and HRCN [39]. The experimental results indicate that on the VesselReID_Adverse dataset, ScA-UniReID with ResNet-50 as the backbone network achieved a mean Average Precision (mAP) of 63.2% and a Rank-1 accuracy of 75.9%, significantly outperforming the baseline CLIP-ReID, which recorded an mAP of 58.1% and a Rank-1 of 70.2%. This demonstrates the superior feature extraction capability of the proposed method in rainy and foggy environments. However, when using ViT-16 as the backbone, although ScA-UniReID still performed well, its mAP was 61.5%, slightly lower than the ResNet-50 version, suggesting that local feature modeling is critical for re-identification tasks in such scenarios.

Furthermore, as illustrated in Table 1, on the Market_Adverse dataset, ScA-UniReID with ResNet-50 achieved an mAP of 80.8% and a Rank-1 accuracy of 92.0%, showing clear improvements over the baseline CLIP-ReID, which obtained an mAP of 79.7% and a Rank-1 of 91.4%. Interestingly, in terms of Rank-1 accuracy, ScA-UniReID was slightly outperformed by ISM [40], possibly due to the more pronounced dynamic pedestrian features in the Market_Adverse dataset, where identity discrimination relies heavily on local detailed information, and ISM may have an advantage in such cases. However, by optimizing cross-modal alignment through scene-aware text prompt learning, ScA-UniReID surpassed ISM in mAP, highlighting its superior ability to handle dynamic degradation factors and effectively separate target features from noise.

In summary, the experimental results demonstrate that, whether using ResNet-50 or ViT-16 as the backbone, ScA-UniReID exhibits significant advantages in person re-identification tasks under complex environments, particularly in the stability of feature extraction.

### 5.3. Ablation Studies

#### 5.3.1. Ablation Study on Scene Encoder Architecture

To systematically validate the effectiveness of the proposed scene encoder architecture, this study conducts ablation experiments comparing three representative structural design schemes: (1) A baseline without control, where the encoder structure is identical to that of the image encoder. The encoder is fine-tuned using only pre-trained weights, and it does not exert any control over the identity encoder. (2) An architecture with a scene encoder but without zero initialization, to investigate the impact of parameter initialization on model performance. (3) The complete architecture proposed in this study, which incorporates the scene encoder along with the zero initialization strategy, aiming to maximize the control over the identity encoder.

This experiment is conducted on both ResNet-50 and ViT-16 backbone networks for cross-architecture validation.

As shown in Table 2, when ResNet-50 is used as the backbone, the CLIP+finetune approach achieves an mAP of 60.6%, while ScA-UniReID w/o zero initialization achieves only 52.5%. This indicates that without zero initialization, the scene encoder may cause instability during early training, thus degrading model performance. In contrast, the proposed ScA-UniReID with zero initialization improves the mAP to 63.2%, representing an improvement of 2.6% over CLIP+finetune and 10.7% over the w/o zero version. This validates the critical role of the zero initialization strategy in enhancing model performance.

When ViT-16 is used as the backbone, CLIP+finetune achieves an mAP of 58.1%, while ScA-UniReID w/o zero achieves 57.3%, again indicating that the absence of zero initialization may negatively affect model stability. The proposed ScA-UniReID based on ViT-16 improves the mAP by 1.7% over CLIP+finetune and by 2.5% over the w/o zero version. Although the improvement is relatively smaller compared to ResNet-50, it still confirms the effectiveness of the proposed method.

In summary, the proposed ScA-UniReID framework achieves the best performance across different backbone networks, demonstrating that the scene encoder can effectively guide the identity encoder to enhance its focus on target regions and improve feature extraction capabilities. This leads to improved accuracy in re-identification tasks under complex environmental conditions. Moreover, the experimental results further validate the necessity of the zero initialization strategy, which helps stabilize the early stages of training and fully unleashes the potential of the scene encoder.

#### 5.3.2. Ablation Study on the Objective Function $L_{control}$

**Figure 5:**
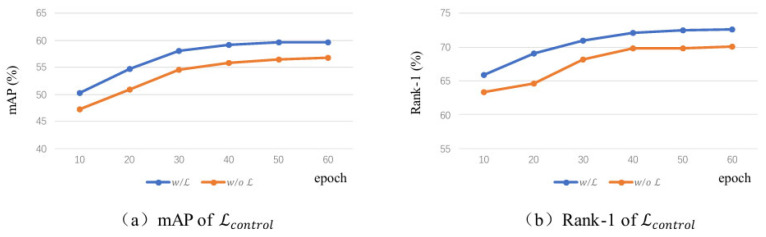
Comparison Chart of Ablation Study on Objective Function $L_{control}$.

To investigate the role of the proposed objective function $L_{control}$ in model training, we conduct an ablation experiment based on the ViT-16 backbone. The performance of two models—one trained with $L_{control}$ (labeled as “*w*/*L*”) and one without it (labeled as “w/o L”)— is compared. As shown in Figure 5, the blue curve (“w/L”) outperforms the orange curve (“w/o L”) on both the mAP and Rank-1 metrics, indicating that $L_{control}$ effectively enhances the model’s recognition capability under rainy and foggy conditions. Specifically, this objective function enables the scene encoder to dynamically perceive degradation features in mixed-degraded images through contrastive learning. It then uses this information as a conditional signal to regulate the attention distribution of the identity encoder, guiding it to focus on identity-related regions. During training, this facilitates the disentanglement of target identity features from noise features, accurately separating the identity information of the target and significantly improving the model’s discriminative ability for degraded images.

#### 5.3.3. Ablation Study on Parameters $\lambda_{1}$ and $\lambda_{2}$

To investigate the impact of different values of parameters $\lambda_{1}$ and $\lambda_{2}$ on the overall objective function $L_{stage2}$ in the second training stage, this study conducts comparative experiments based on the ViT-16 backbone, setting different weight combinations for $\lambda_{1}$ and $\lambda_{2}$.

As shown in Table 3, although varying the parameter weights leads to some fluctuations in performance metrics such as mAP and results at different k-values, the overall model performance remains robust. This indicates that the proposed framework exhibits strong generalization with respect to parameter selection. It can achieve satisfactory target re-identification performance without requiring precise parameter tuning, further demonstrating the framework’s good adaptability and stability.

### 5.4. Visualization Results

#### 5.4.1. Retrieval Performance Comparison

**Figure 6:**
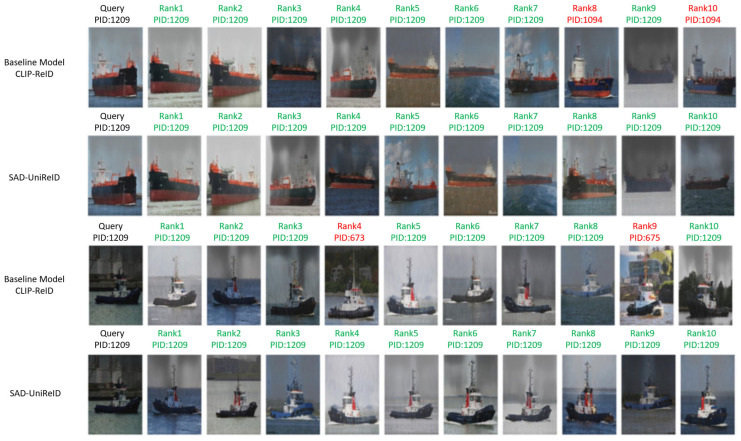
Comparison of Retrieval Results Between the Proposed ScA-UniReID Method and the Baseline Model CLIP-ReID.

To intuitively demonstrate the retrieval capability of ScA-UniReID under rainy and foggy conditions, this study conducts visualization analysis on the *VesselReID_Adverse* test set. Specifically, query images along with their top-10 matching results are selected and displayed. The retrieval results are shown in Figure 6.

ScA-UniReID exhibits strong target recognition ability under complex weather conditions such as rain and fog. In contrast, the baseline model shows clear limitations under the same conditions. The visualization results indicate that, in the presence of rain, haze, and wave interference, the baseline model struggles to effectively separate target features from noise, leading to loss of detailed vessel information and degradation in retrieval performance. For example, in the first case, the baseline model is significantly affected by rain interference and fails to capture key structural features, resulting in incorrect matches with targets of similar color but different identity.

In comparison, ScA-UniReID successfully identifies the correct target under the same challenging conditions, validating its strong adaptability to adverse weather environments. This advantage is primarily attributed to the introduction of the scene encoder, which actively learns noise characteristics from degraded regions and optimizes the attention distribution of the identity encoder. As a result, the model remains focused on target-related features even under complex disturbances such as rain and fog.

#### 5.4.2. Feature Visualization

To further verify the feature learning capability of ScA-UniReID under degraded conditions, Figure 6 illustrates a comparison between the target feature attention regions of ScA-UniReID and the baseline method CLIP-ReID in rainy and foggy scenarios.

**Figure 7:**
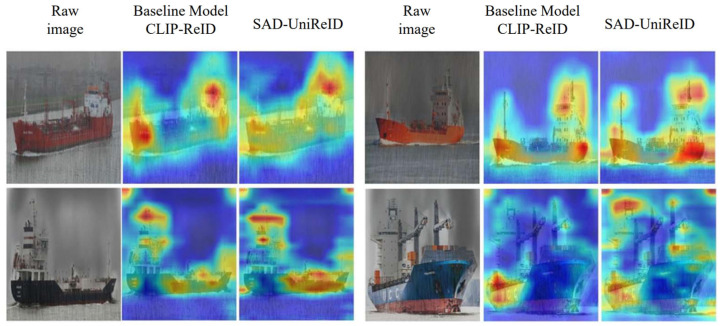
Feature Attention Comparison Between ScA-UniReID and CLIP-ReID Under Rainy and Foggy Conditions.

From the feature visualization results, it can be observed that CLIP-ReID primarily focuses on local high-salience areas of the vessel, such as the bow, stern, and mast, which are structurally prominent parts. as illustrated in Figure 7. However, this localized focus strategy has clear limitations under degraded conditions. When parts of the target are affected by rain or fog noise, the model struggles to form stable global features, leading to incomplete target representation and thus affecting retrieval accuracy.

In contrast, guided by the scene encoder, ScA-UniReID is capable of capturing the overall structure and key identity features of the target more comprehensively. Heatmaps show that ScA-UniReID not only extracts local high-salience features but also evenly distributes attention across the entire outline, hull shape, and distinctive details, ensuring the completeness of the target features. Additionally, under degraded conditions, the scene encoder effectively reduces the model’s focus on degradation features, allowing the identity encoder to concentrate more on identity-related features.

Overall, relying on the scene-aware mechanism, ScA-UniReID enhances the global perception ability of target features, maintaining stable feature representations in rainy and foggy environments, effectively reducing environmental interference. This further validates its advantage in improving recognition robustness.

#### 5.4.3. Robustness Analysis Under Adverse Weather Degradations

**Figure 8:**
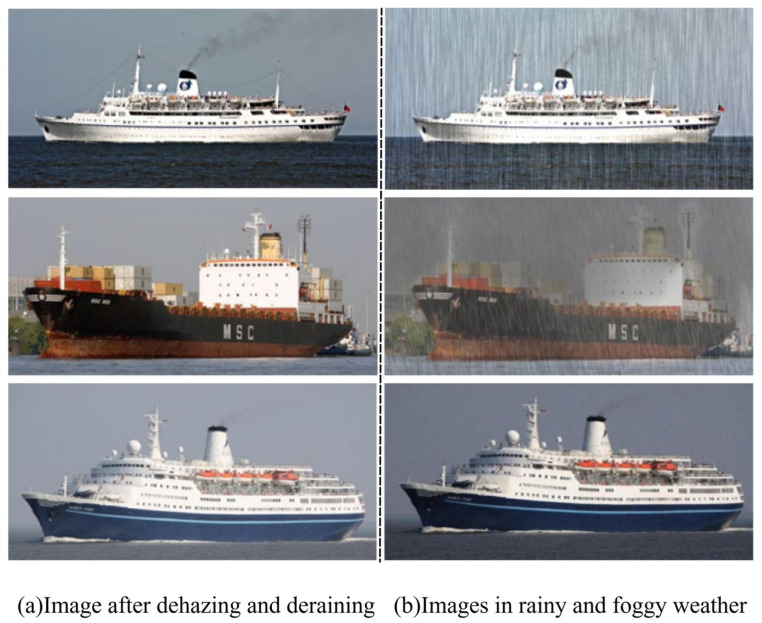
Qualitative Comparison of Degraded and Restored Images under Adverse Weather Conditions.

In complex environments such as rain and haze, target re-identification faces significant challenges, primarily due to visual information degradation and the superposition of multiple interfering factors. As illustrated in Figure 8b, rainfall introduces randomly distributed rain streaks that occlude key regions of the target, leading to the loss of fine-grained details (first row). Meanwhile, the rain–haze effect reduces image contrast, blurring object contours and textures and thereby weakening discriminative capability (second row). In addition, overcast low-light conditions result in insufficient illumination and increased noise, further affecting the stability of feature extraction (third row). More importantly, these degradation factors often interact and couple with each other, substantially increasing the difficulty of recognition in real-world scenarios and posing higher demands on the robustness and generalization ability of ReID models.

To mitigate these issues, As illustrated in Figure 8a. existing studies often introduce image restoration techniques such as dehazing and deraining as a preprocessing step to improve visual quality. These methods help suppress degradation artifacts, enhance image clarity, and recover structural details, thereby providing more reliable inputs for subsequent feature extraction. However, such approaches mainly focus on low-level visual enhancement and may not fully preserve identity-related discriminative features, which are crucial for robust re-identification under complex degradation conditions.

#### 5.4.4. t-SNE Visualization

**Figure 9:**
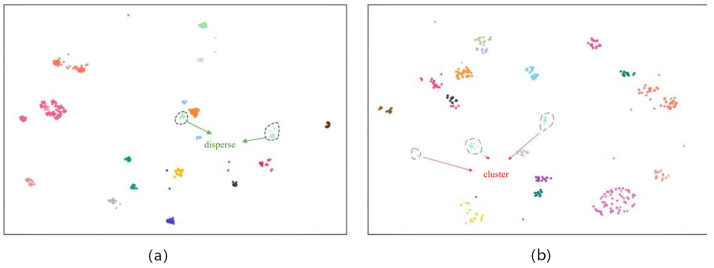
(**a**) t-SNE Visualization of the Proposed ScA-UniReID Method; (**b**) the Baseline Model CLIP-ReID.

To further demonstrate the effectiveness of ScA-UniReID, this section employs the t-SNE visualization method [18], as shown in Figure 9. This analysis compares the feature distribution in the latent space between the baseline model and the proposed method in the second training stage, using 20 randomly selected classes from the dataset.

In Figure 9a, the feature distribution of CLIP-ReID appears scattered, with sample points from the same class spreading over a large area, indicating poor clustering within the same category. Different colors are used to distinguish individual samples for visualization purposes only and do not correspond to specific semantic categories. This suggests that the baseline model struggles to learn stable identity representations under degraded conditions, leading to reduced feature discriminability.

In contrast, as shown in Figure 9b, the features generated by ScA-UniReID exhibit much better intra-class compactness and inter-class separability. The feature points within the same category are more tightly clustered, while different categories are clearly distinguished from each other. Although colors are randomly assigned for visualization, the clustering structure clearly demonstrates improved feature discrimination. This indicates that the proposed method can more accurately extract identity features in rainy and foggy scenarios and effectively differentiate between different classes.

## 6. Conclusions

This paper presents a novel scene-aware person re-identification approach to confront the fundamental challenge of image degradation arising from the coupled effects of rain, fog, and other adverse factors in real-world scenarios. In practice, severe weather dramatically reduces image contrast and blurs structural details, leading to drastic shifts in the feature space and a sharp decline in model generalization. To systematically alleviate these issues, we intervene at two complementary levels—feature modeling and semantic guidance. First, a dedicated scene encoder together with scene-aware prompt tokens is introduced to inject environmental priors into the encoding stage, enabling the model to dynamically perceive and compensate for rain-and-fog-induced degradation. Second, a contrastive-learning objective between the identity encoder and the scene encoder is employed to explicitly constrain the distributions of target features and environmental noise in the feature space, achieving effective disentanglement. Extensive experiments demonstrate that the proposed ScA-UniReID not only yields significant accuracy improvements under diverse adverse-weather settings but also exhibits strong generalization to previously unseen conditions.

## Figures and Tables

**Table 1:** Results of State-of-the-Art Methods on the VesselReID_Adverse and Market_Adverse Datasets (bold indicates the best performance).

Tecnology	Conference/ Journal’Year	VesselReID_Adverse		Market_Adverse	
		mAP	Rank-1	mAP	Rank-1
AWG	TPAMI’21	51.2	64.8	75.9	89.9
HRCN	ICCV’21	53.9	66.3	72.1	88.0
CIL	NeurIPS’21	54.4	68.5	68.7	85.4
ISM	ICME’21	54.8	68.3	79.2	**92.2**
TransReID	ICCV’21	54.8	68.0	78.7	90.4
RotTrans	ACMMM’22	53.6	66.1	76.7	89.2
SJDL	AAAI’22	56.4	69.8	63.4	83.0
PHA	CVPR’23	52.5	65.5	76.2	88.7
CLIP-ReID (ViT-16)	AAAI’23	57.6	70.9	78.8	89.9
CLIP-ReID	AAAI’23	61.2	73.3	79.7	91.4
DenoiseRep	NeurIPS’24	53.2	68.0	79.8	89.9
DCCC	ICASSP’24	47.3	63.4	-	-
DHCCN	TCSVT’24	36.8	56.0	-	-
CCL	IJCNN’25	50.5	66.4	-	-
ScA-UniReID (ViT-16)	-	59.8	71.8	**81.2**	91.6
ScA-UniReID	-	**63.2**	**75.9**	80.8	92.0

**Table 2:** Comparison Results of Different Scene Encoder Architectures on the VesselReID_Adverse Dataset.

Backbone	Tecnology	mAP	k = 1	k = 5	k = 10
	CLIP+fineune	60.6	73.3	89.9	93.6
ResNet-50	ScA-UniReID w/o zero	52.5	69.8	87.8	92.2
	ScA-UniReID(ours)	63.2	75.9	91.0	94.6
	CLIP+finetune	58.1	70.8	88.5	92.8
ViT-16	ScA-UniReID w/o zero	57.3	69.9	87.1	92.5
	ScA-UniReID(ours)	59.8	71.8	88.7	93.2

**Table 3:** Comparison Results of Different Weight Settings on the VesselReID_Adverse Dataset.

$\lambda_{1}$	$\lambda_{2}$	mAP	k = 1	k = 5	k = 10
1	1	59.8	71.8	88.7	93.2
1	2	59.8	72.2	88.7	93.2
1	3	59.4	72.3	88.3	92.9
2	2	59.7	72.5	88.6	92.7
3	1	59.3	73.3	88.4	92.9

## Data Availability

The original contributions presented in the study are included in the article, further inquiries can be directed to the corresponding author.

## Abbreviations

The following abbreviations are used in this manuscript:

ReID	Re-identification
ScA	Scene-Aware Degradation
CLIP	Contrastive Language-Image Pretraining
VLP	Vision-Language Pretraining
